# European medicines agency guideline for biological medicinal products: a further step for a safe use of biosimilars

**DOI:** 10.1186/s12948-015-0010-3

**Published:** 2015-05-15

**Authors:** Carlo Agostini, Giorgio Walter Canonica, Enrico Maggi

**Affiliations:** Department of Medicine, University of Padua, Padua, Italy; University of Genoa, IRCCS AOU San Martino-IST, Genoa, Italy; Department of Clinical and Experimental Medicine, University of Florence, Florence, Italy

Allergists and clinical immunologists welcome the recent revision of the guideline on similar biological medicinal products containing biotechnology-derived proteins (“biosimilar”) that the European Medicines Agency (EMA) has published on December 18, 2014 [1]. In the past justifiable doubts on an uncritical use of biosimilars have been formulated by most scientific societies, including our Italian Society of Allergy, Asthma and Clinical Immunology (SIAAIC). Perplexities were linked to the fact that relatively small changes in manufacturing, characterization and/or formulation of a biological medical product can significantly alter its efficacy, safety and/or immunogenicity [2]. This is particularly relevant when we consider the possible use in clinical practice of biosimilar monoclonal antibodies. Indeed, the previous EMA guidelines addressed manufacturing, not clinical pharmacology, toxicology, pharmacokinetic, pharmacodynamic and clinical considerations.

It is sufficient to consider how changes in the cell line that produce the monoclonal antibody and/or post translational modifications, including glycosylation patterns, could alter the specificity of the target antigen binding and the effector functions of the new biosimilar. For instance, the drug cytotoxic activity or those functions that are mediated by FcγR interactions may be in theory altered by the culture conditions. Minimal changes in the production process of the biosimilar could also result in cross linking and formation of aggregates which have been described to activate B cells in T-cell independent way. Furthermore, small alterations in the manufacturing process might impact not only the half-life of the product but also its affinity and avidity, or its dose-response efficacy. Obviously, there are advantages in using biosimilars and the economic savings is considered the most important, in particular for those national health systems that are at risk of being drained by the constant increase in the cost of the many biologic drugs that every year are introduced in the clinical practice.

The fact that biological drugs are so susceptible to variation suggests the importance of an efficient control. It is mandatory to be sure that pharmaceutical companies produce a version of the drug that is adequately similar to the approved branded product [3]. The current version of the EMA guideline represents a real advancement in this context since it details a number of requirements that biopharmaceutical companies must follow to navigate the approval process and to delineate the quality standards for reproducing such medications. In particular, for a biosimilar that enter the process of marketing authorization, the EMA guideline recommends a stepwise conduct of non-clinical and clinical studies and dictates a number of crucial steps to be followed before authorization, including the use of pharmacodynamic and pharmacokinetic studies, clinical trials of appropriate patient populations, the choice of clinical endpoints in efficacy trials, the development of clinical safety studies, an evaluation of the immunogenicity of the biosimilar, and pharmacovigilance studies aimed at extrapolate safety and efficacy of the new biosimilar with respect to the already authorized branded medicinal product.

To move more in depth on to the core of the new EMA guideline, the Agency recommends that relevant non-clinical studies in support of biosimilarity should be planned before initiating any clinical trial in humans. These studies should be based on a series of distinct approaches that should mainly evaluate *in vitro* comparative assays between the original product and the biosimilar. These *in vitro* test should be aimed at evaluating binding capability to targets as well as signal transduction and functional/viability activity of the products that are compared. In addition, the presence of potentially relevant differences between the two products should be considered in terms of formulation, quality and stability of the drugs. Finally, *in vivo* animal studies should be planned when *in vivo* data are mandatory to be able to proceed to clinical trials.

Before approval EMA requires the development of clinical trials that should be able to generate findings clearly supporting the biosimilar comparability between the two products under evaluation. Clinical studies should include pharmacokinetics and, if required, pharmacodynamic comparisons between the already approved drug and the new biosimilar. Furthermore, any biopharmaceutical company that proposes the introduction of a new biosimilar should demonstrate comparable clinical efficacy between the reference drug and new biosimilar medicinal product that is under evaluation. The drug efficacy should be tested using adequately powered, randomized, parallel group comparative clinical trial(s), preferably double-blind, that should be designed to obtain clear efficacy endpoints. As a part of clinical efficacy studies comparative safety data must be collected by the investigators and provided to the Agency in an adequate dossier.

Another important principle introduced by EMA is the need of an assessment of potential immunogenicity of monoclonal antibodies or, in general terms, of those therapeutic proteins that are investigated in a comparative manner versus a determined reference product. The Agency recommends the standardization of analytical assays, that should be capable of detecting antibodies against both the biosimilar and the reference molecule; in any case, the test should at least be able to detect such antibodies that are developed against the biosimilar product. In case of drug that are supposed to be used in regimens of chronic administration (for example in the event of drugs used for chronic inflammatory disorders), the Agency requires one-year pre-authorization follow up in order to detect possible late sensitization to the biosimilar. If there are data on a possible immunogenicity of the drug, an additional period (up to one-year) could be required in the post-marketing authorization phases.

In this last regard, the previous clinical experiences on the use of biologics in human diseases have clearly shown that data from pre-authorization clinical studies not always allow the recognition of all adverse effects. For instance, immunogenicity of biologics has been often documented only after years of follow-up in patients with different human diseases. Noteworthy, a significant immunogenicity was sometimes observed only in the context of a selective therapeutic indication of the drug. Taken together these considerations [4], it is clear that during the post-approval phases clinical safety of biosimilars should be monitored closely and we believe that an active pharmacovigilance role could be at least in part played by the Members of the Medical Scientific Societies who are preparing for the use in clinical practice, including the Members of the Italian Society of Allergology, Asthma and Clinical Immunology.

The patent expiration of the biotech product will witness the appearance of new biosimilars in the next few years: indeed three patents have been expired in 2012, nine in 2013, seven in 2014 and sixteen will expire in the current year [5]. For this reason it is easy to anticipate that in order to favor the necessary containment of health care costs physicians involved in allergic and immunologic diseases as well as reumathologists, neurologists, gastroenterologists, dermatologists or oncologists will be encouraged by the majority of local health administrators to use biosimilars (which will offer a potential reduction of 30% of costs of the reference products) [6]. Who more than an allergist can take the responsibility to assess immunogenicity potential of biosimilars during their use in real life and to check the *in vivo* sensitization by validated skin tests? Who more than clinical immunologists and allergists have the expertise to propose new specific assays to compare the immunogenicity of biosimilars vs biotherapeutics or to plan novel desensitization protocols? The supposed date for coming in effect of the Guideline is July 1st 2015; allergists and clinical immunologists should be prepared for that data.
